# MST4: A Potential Oncogene and Therapeutic Target in Breast Cancer

**DOI:** 10.3390/cells11244057

**Published:** 2022-12-15

**Authors:** Ritu Arora, Jin-Hwan Kim, Ayechew A. Getu, Anusha Angajala, Yih-Lin Chen, Bin Wang, Andrea G. Kahn, Hong Chen, Latif Reshi, Jianrong Lu, Wenling Zhang, Ming Zhou, Ming Tan

**Affiliations:** 1 Mitchell Cancer Institute, University of South Alabama, Mobile, AL 36604, USA; 2 Markey Cancer Center, Department of Toxicology and Cancer Biology, University of Kentucky, Lexington, KY 40508, USA; 3 Graduate Institute of Biomedical Sciences, China Medical University, Taichung 406040, Taiwan; 4 Department of Physiology, School of Medicine, College of Medicine and Health Sciences, University of Gondar, Gondar P.O. Box 196, Ethiopia; 5 Department of Mathematics and Statistics, University of South Alabama, Mobile, AL 36688, USA; 6 Department of Pathology, School of Medicine, University of Alabama at Birmingham, Birmingham, AL 35294, USA; 7 Department of Biochemistry & Molecular Biology, College of Medicine, University of Florida, Gainesville, FL 32610, USA; 8 Department of Laboratory Medicine, Xiangya School of Medicine, Central South University, Changsha 410013, China; 9 Cancer Research Institute, School of Basic Medical Sciences, Central South University, Changsha 410013, China; 10 Research Center for Cancer Biology, China Medical University, Taichung 406040, Taiwan

**Keywords:** MST4, EMT, AKT, apoptosis, breast cancer

## Abstract

The mammalian STE 20-like protein kinase 4 (MST4) gene is highly expressed in several cancer types, but little is known about the role of MST4 in breast cancer, and the function of MST4 during epithelial-mesenchymal transition (EMT) has not been fully elucidated. Here we report that overexpression of MST4 in breast cancer results in enhanced cell growth, migration, and invasion, whereas inhibition of MST4 expression significantly attenuates these properties. Further study shows that MST4 promotes EMT by activating Akt and its downstream signaling molecules such as E-cadherin/N-cadherin, Snail, and Slug. MST4 also activates AKT and its downstream pro-survival pathway. Furthermore, by analyzing breast cancer patient tissue microarray and silicon datasets, we found that MST4 expression is much higher in breast tumor tissue compared to normal tissue, and significantly correlates with cancer stage, lymph node metastasis and a poor overall survival rate (*p <* 0.05). Taken together, our findings demonstrate the oncogenic potential of MST4 in breast cancer, highlighting its role in cancer cell proliferation, migration/invasion, survival, and EMT, suggesting a possibility that MST4 may serve as a novel therapeutic target for breast cancer.

## 1. Introduction

Breast cancer is the most frequently diagnosed cancer (2.26 million cases in 2020), and one of the top five leading causes of cancer-related death worldwide (685,000 deaths) (www.who.int/news-room/fact-sheets/detail/cancer, accessed on 14 December 2022). Although we have made tremendous strides towards earlier detection and treatment of breast cancer, advanced stage and metastatic disease continues to result in ultimate cancer death.

Epithelial–mesenchymal transition (EMT) is a major developmental process involving a cascade of biological events in which epithelial cells lose their cell polarity and cell–cell adhesion and gain invasive properties transforming into mesenchymal-like cells. EMT events are involved in essential processes, such as tissue development (embryogenesis, gastrulation, and neural crest formation) and post-injury restoration (tissue regeneration and wound healing). However, EMT is also known to represent some of the hallmarks of malignancy, including invasion and metastasis [1,2,3]. EMT plays a key role in the initiation of cancer metastasis. During malignant tumor formation, EMT regulatory pathways controlling cellular adhesion and the polarization of the cell/cytoskeleton change, leading to cellular detachment, migration, intra-/extravasation, and finally metastasis [3,4]. Typically, cancer cells transform from an epithelial to mesenchymal phenotype by decreasing expression of epithelial markers such as E-cadherin and increasing factors such as N-cadherin, vimentin, snail, slug, and other recognized cellular proteases [5,6,7]. Additionally, multiple oncogenic signaling pathways mediated by peptide growth factors, Src, Ras, Ets, integrins, Wnt/β-catenin, Notch, ERK, and AKT have all been associated with EMT [8,9,10,11,12,13].

The AKT gene is involved in many cellular responses that range from cell proliferation, survival, metabolism, and other cellular activities [14,15,16,17]. It is frequently activated in human epithelial cancers, with AKT2 gene activation in ovarian carcinoma linked to aggressive clinical behavior and a loss of the histological features of epithelial differentiation [18,19]. Studies in squamous cell carcinoma overexpressing activated AKT demonstrated that EMT was regulated by AKT activation [20]. These findings are consistent with many other studies showing that AKT directly affects epithelial-mesenchymal transition and subsequently epithelial cell morphology changes, tumorigenicity, cell motility and tumor cell invasiveness. 

The mammalian STE 20-like protein kinase 4 (MST4) gene, also known as serine/threonine protein kinase 26 (STK26), is one of three proteins identified within the germinal center kinase (GCK) subfamily III. It consists of MST3, MST4, and STK25 that are located upstream of the MAPK-related kinase [21,22]. This protein subfamily shares a conserved kinase domain, with a different carboxyl terminal domain that mediates homo- and hetero-dimerization with the adaptor protein CCM3 [22,23]. In human tissue, MST4 is highly expressed in the thymus, placenta, lymphocytes, and leukocytes [22,24]. MST4 kinase exhibits multiple roles in the regulation of signaling pathways that govern cell mitosis, homeostasis, polarity, migration, apoptosis, proliferation, and cellular differentiation [24,25,26,27,28,29]. Our previous research has identified MST4 as a direct target of miRNA 4728 in human breast cancer [21]. However, beyond this, very little is known about the role and involvement of MST4 in breast cancer. 

In this study, we report an unrecognized function of MST4 for EMT regulation via the MST4-AKT pathway and oncogenic potential of MST4 in breast cancer, together with its function in promoting cell growth, migration, and invasion, suggesting that MST4 may serve as a potential novel oncogene for breast cancer.

## 2. Materials and Methods

### 2.1. Cell Lines and Transfection

Human breast cancer cell lines MDA-MB-231 and BT474, and human embryonic kidney HEK293T cells were purchased from American Type Culture Collection (ATCC, Manassas, VA, USA). Cells were cultured in DMEM/F-12 (Mediatech Inc., Manassas, VA, USA) supplemented with 10% FBS (R&D Systems, Inc. Minneapolis, MN, USA) at 37 °C, 5% CO^2^. Cells were routinely tested for mycoplasma contamination. Lipofectamine 2000 (Thermo Fisher Scientific, Waltham, MA, USA) was used for transfection according to the manufacturer’s instructions. After transfection for 48 h, cells were harvested and prepared for immunoblot.

### 2.2. Generation of Stable Cell Lines

To generate stable MST4 knockdown cell lines, lentivirus particles were prepared by transfecting HEK293T cells with scrambled (scr) or shMST4 plasmids (Santa Cruz Biotechnology, Dallas, TX, USA). For lentivirus transduction, MDA-MB-231 and BT474 cells at approximately 80% confluency were infected with lentivirus-bearing specific scr or shRNAs in growth medium containing 8 µg/mL polybrene for 24 h. The infected cells were sub-cultured for 3 days in growth medium containing 2 µg/mL of puromycin. The scrambled (scr) and shMST4 plasmid transfected cell pools were subjected to Western blot analysis to confirm MST4 expression, and the cells were used for the later experiments.

### 2.3. MST4 Wild Type and MST4 Kinase Defective Mutant Stable Cell Line Construction

MST4 WT and kinase defective mutant (T178A) were kindly provided by Dr. Wierman [30]. MDA-MB-231 cells were transfected with plasmid DNA and after 48 h, the cells were harvested for Western blot analysis. For stable MST4 gene expression, transfected cells with MST4 and MST4 T178A were incubated for 2 weeks in media containing 200 μg/mL G418. High MST4 expression in cells was evaluated with Western blot analysis.

### 2.4. Biological Phenotype Assays 

Cell proliferation was assessed using direct cell counting for every 24 h from days 1 to 4. Migration and invasion assays were performed using a 24-well format and Boyden chambers coated without or with Matrigel matrix (BD Pharmigen, Franlin Lakes, NJ, USA). Briefly, 5 × 10^4^ MDA-MB-231 (pcDNA/MST4 and scr/shMST4) and BT474 (scr/shMST4) cells were seeded in the top wells. At 8 h (for migration assay) and 24 h (for invasion assay), cells that invaded through the Matrigel matrix and/or 8 μm pore membrane were fixed with 4% paraformaldehyde, stained with 0.05% crystal violet, and counted under a light microscope. Three-dimensional (3D) cell culture growth was assessed for MDA-MB-231 (pcDNA/MST4 and scr/shMST4) and BT474 (scr/shMST4) cells according to the published protocol [21]. The membrane-permeant JC-1 dye (Invitrogen MitoProbe JC-1 Assay Kit, M34152) was used in apoptosis studies to monitor mitochondrial membrane potential.

### 2.5. EMT Gene PCR Array

The human EMT gene RT^2^Profiler™ PCR array (Qiagen, Germantown, MD, USA) was used to examine the EMT related gene profiling. Total RNA was extracted, and the cDNA produced was diluted in PowerTrack SYBR green master mix (Thermo Fisher Scientific, Waltham, MA, USA) and added to the 96-well qPCR plate containing primers for each of the genes of interest and the control housekeeping genes. Data analysis was conducted using the online Qiagen Gene Globe platform (https://geneglobe.qiagen.com/gb/analyze/ accessed on 22 March 2017) which calculated 2^−ΔCt^; fold change values for each gene are based on the values of the housekeeping genes included in the kit., cDNA synthesis and real time PCR.

Total RNA was extracted from MDA-MB-231, BT474 scr and shMST4 cells using Trizol reagent (Invitrogen, Waltham, MA, USA) according to the manufacturer’s instructions. Total RNA (2 μg) was reverse-transcribed using a high capacity cDNA Reverse Transcription Kit (Applied Biosystems, Waltham, MA, USA) according to the manufacturer’s instructions. Prepared cDNA was diluted and used for the EMT PCR array (Qiagen). A few genes selected from the array results were analyzed via qPCR in BT474 (scr/shMST4) cells using previously published primers, snail, forward 5′-TACCTTCCAGCAGCCCTACG-3′, reverse 5′-AGCCTTTCCCACTGTCCTCAT-3′, slug.

### 2.6. Western Blot Analysis

The cells were collected and lysed using NP-40 lysis buffer with proteinase and phosphatase inhibitors. The total protein concentration was measured using BCA solution (Pierce, Rockford, IL, USA). The samples were separated with SDS-PAGE (Bio-Rad Laboratories, Inc., Hercules, CA, USA) and transferred to PVDF membranes (Millipore, Burlington, MA, USA). Following blocking with 5% non-fat milk in TBS, the membranes were probed with antibodies at 4 °C overnight, and blots were washed three times for 15 min. The membrane was incubated with HRP-conjugated secondary antibody (mouse or rabbit) (Cell signaling technology, Danvers, MA, USA) for 1 h. The protein bands of interest were visualized using a chemiluminescence kit (Millipore, Burlington, MA, USA). The band intensity was quantified using Image lab software (Bio-Rad Laboratories, Inc., Hercules, CA, USA).

### 2.7. Tissue Microarray Analysis

Human breast cancer TMAs were purchased from US Biomax (Rockville, MD, USA) and Novus Biologicals (Littleton, CO, USA). TMA slides were processed with a series of steps that included dewaxing, hydration, antigen retrieval, and staining. A semi-quantitative scoring criterion was used in which the staining intensity was recorded. To achieve the staining index, each core was scored based upon the intensity of the staining, scored as negative = 0, weak positive = 1, moderate positive = 2, and strong positive = 3. Overall survival was defined as the time of initial diagnosis until the date of death. 

### 2.8. TCGA Data Analysis

BRCA Illumina, high throughput sequencing RNA seq data (level 3 gene expression data) for BRCA solid tumors and normal tissue samples were downloaded from the TCGA data portal (The Cancer Genome Atlas), originally sequenced and submitted by www.unc.edu (accessed on 13 January 2017). FPKM_UQ data files were obtained, merged, and normalized using R program. The data files examined the MST4 gene (NCBI Gene ID# 51765, Ensemble ID ENSG00000134602) for 1102 tumors and 113 normal samples. Clinical information data were also downloaded and organized based on receptor status (Luminal (ER positive and/or PR positive), HER2 type (ER negative, PR negative and HER2HER2 positive), triple negative breast cancer (ER, PR and HER2 negative, TNBC)) or by breast cancer stage. MST4 gene expression data was obtained for primary tumor samples. Quantile (below Q1 as MST4 low and above Q3 MST4 as high levels) was used as the cutoff and grouped as MST-4 high or low samples. Lymph node counts were obtained for these samples and compared. The t-test (2-tailed distribution and non-parametric unequal variance) was performed between two groups and the *p*-value was obtained, with all data plotted utilizing R software. We evaluated the prognostic significance of MST4 using the KM plotter (http://kmplot.com/) (accessed on 13 January 2017). Patient data sources for the database include TCGA and GEO. The correlation between MST4 mRNA expression levels and overall survival (OS) rate were calculated via the Kaplan–Meier curve and log-rank test. The results were shown via Kaplan–Meier survival plots. Hazard ratio (HR) and 95% confidence were calculated automatically with the website tool. The values of each group are shown as the mean ± SD; *p*-value < 0.05 was regarded as statistically significant.

### 2.9. Statistical Analysis

All the experiments were independently performed at least three times, with the data presented as the mean (±standard error mean). All data were analyzed and presented using GraphPad Prism (GraphPad Software Inc., San Diego, CA, USA, version 7.0) and Excel software. Statistical significance of difference was calculated using a student’s T-test with significant differences defined as at least a *p*-value of <0.05. 

## 3. Results

### 3.1. MST4 Increases Breast Cancer Cell Growth

**Figure 1:**
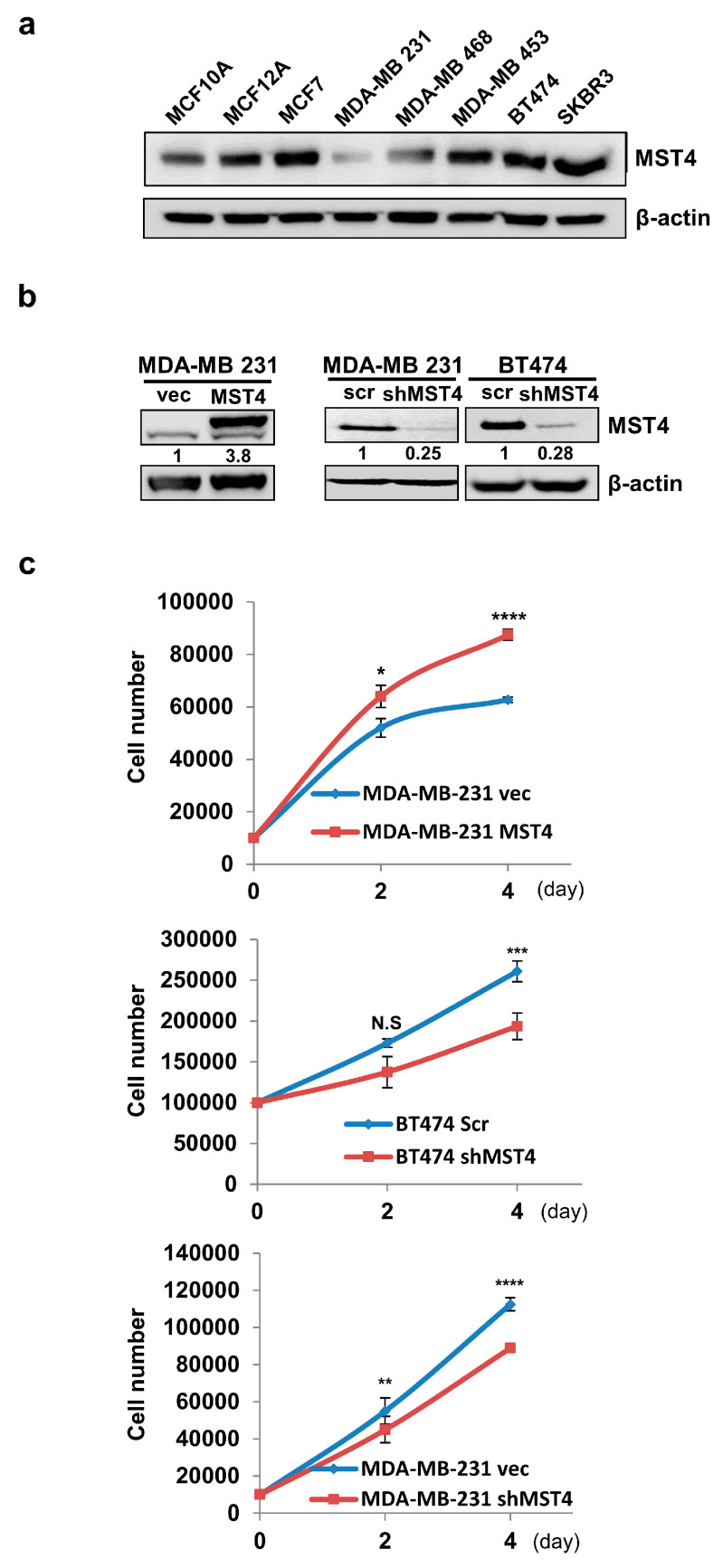
MST4 increases breast cancer cell growth. (**a**) MST4 expression level detected in different breast cancer cell lines. (**b**) MST4 expression level increased after overexpression and reduced up to 70–80% after knocking down MST4. (**c**) MST4 silencing reduces cell growth and overexpression increases cell growth after 4 days of transfection in breast cancer cells. MDA-MB-231 and BT-474 cell lines were grown up to 80% confluency and infected with scr or shMST4 lentivirus particles, and puromycin (2 μg/mL) was used for selection. The error bars represent SEM. * indicates *p* value difference between shMST4 and scrambled or MST4 overexpression and vector cells at *p* < 0.07 (*), *p* < 0.03 (**), *p* < 0.005 (***), and *p* < 0.001 (****) and levels by student’s *t*-test. N.S. indicates no statistical significance (*p* > 0.05). After 2–3 passages, cells were collected to check MST4 expression levels through Western blot. β-actin was a loading control. Data are evaluated through at least three independent experiments.

MST4 has been reported to be upregulated in many cancer types. However, little is known for the role of MST4 in breast cancer. To determine the role of MST4 in breast cancer, the level of MST4 expression was analyzed in six human breast cancer cell lines—MCF7, MDA-MB-231, MDA-MB-468, MDA-MB-453, BT-474, and SKBR3—and two non-cancerous cell lines, MCF10A and MCF12A (Figure 1a). MST4 was highly expressed in all breast cancer cell lines except in MDA-MB-231. To identify the role of MST4 in cancer cell growth and proliferation, we generated MST4 overexpression in MDA-MB-231 cells and shRNA knockdown cell lines in MDA-MB-231 and BT-474 cells. The overexpression and down-regulation of MST4 levels in these cells were verified via Western blot analysis (Figure 1b). We then assessed the effect of MST4 expression on cell growth by direct cell counting. MST4 overexpressing MDA-MB-231 cells grew faster compared to the vector control. However, knockdown of MST4 in MDA-MB-231 and BT-474 cells significantly attenuated the rate of cell growth (Figure 1c). These results indicate that MST4 positively influences the proliferation of breast cancer cells. 

### 3.2. MST4 Promotes the Invasive Properties of Breast Cancer Cells

**Figure 2:**
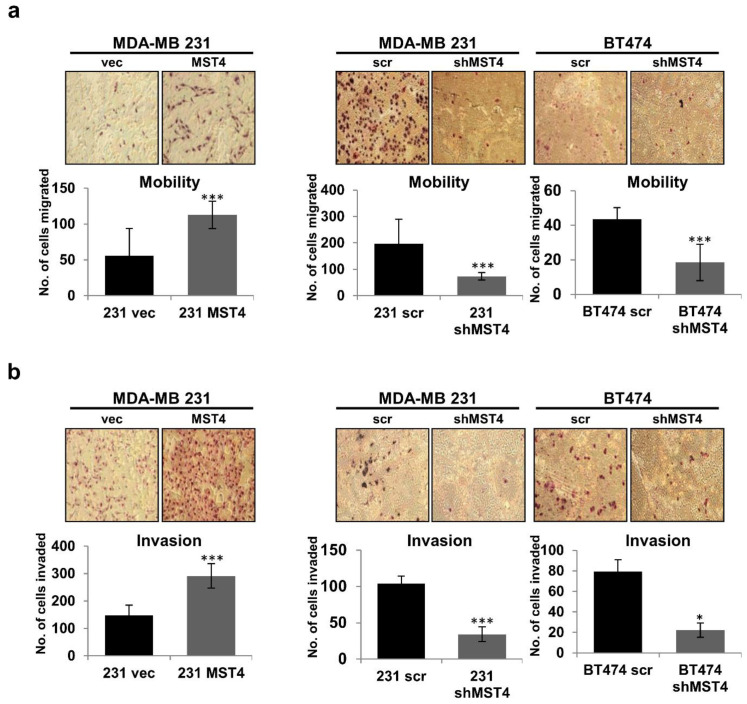
MST4 promotes the invasive properties of breast cancer cells. MDA-MB-231 scr/MST4/shMST4 and BT-474 scr/shMST4 cells were seeded on non-coated or matrigel-coated membranes for (**a**) motility and (**b**) invasion assays, respectively, and incubated for 24 h. DMEM containing 10% fetal bovine serum in the lower chamber was used as a chemo-attractant. The histograms show the mean of three independent experiments, and the error bars represent SEM. * indicates statistically significant difference between shMST4 and scrambled cells at *p* < 0.05 (*), and *p* < 0.001 (***) levels by student’s *t*-test. The magnification for both migration and invasion assays was 100×).

Increased cancer cell mobility and invasiveness are crucial consequences of cancer cell EMT [3]. We examined the role of MST4 in breast cancer cell migration and invasiveness. In motility and invasion assays, chemo-attractive cell mobility and invasiveness of the MST4 overexpressing cells were significantly increased but attenuated when the level of MST4 was knocked down by shRNA (Figure 2a,b). The much larger ranges of change in cell migration and invasion could not be explained by the marginal ranges of change in cell proliferation induced by MST4 within the same time frame (Figure 1), indicating that MST4 promotes the intrinsic invasive potential of these cancer cells. Together, these data show that overexpression of MST4 can enhance cell growth and invasiveness, while knocking down MST4 gene expression can reduce cell growth, migration, and invasion capacity of breast cancer cells. 

### 3.3. Activation of MST4 Is Required for Breast Cancer Cell Survival and Enhances Chemoresistance

**Figure 3:**
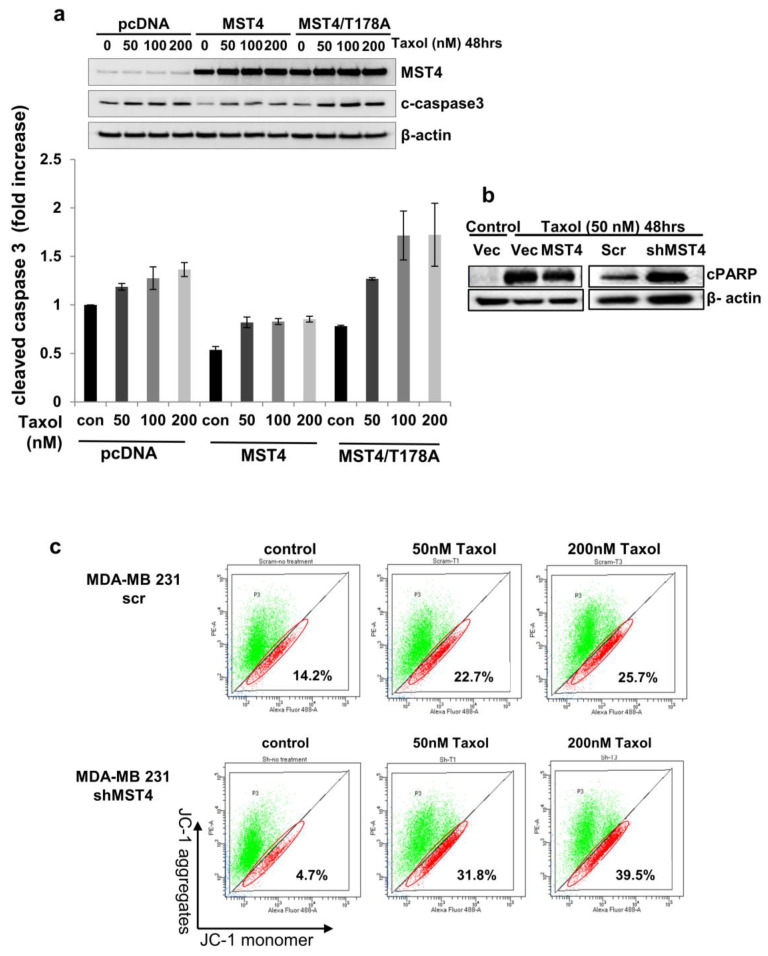
Activation of MST4 is required for cancer cell survival and enhances chemoresistance. (**a**) Cells were treated with 50 and 200 nM Taxol for 48 h and analyzed via immunoblot. PARP cleavage was evaluated from quantification of total PARP expression level. β-actin was a loading control. Data are evaluated through at least three independent experiments. Total protein was isolated from MST4 knockdown MDA-MB-231 and BT-474 cells and subjected to immunoblot. (**b**) Western blot of PARP cleavage in MDA-MB-231 cells with different expression levels of MST4. Exogenous expression of MST4 inhibited Taxol-induced PARP cleavage, while knockdown of MST4 increased Taxol-induced PARP cleavage in MDA-MB-231 cells. (**c**) Cells were treated with 50 and 200 nM Taxol for 48 h and checked for mitochondrial apoptosis through flow cytometry. JC-1 staining shows reduction in mitochondrial membrane potential (Ψ m) in cancer cells treated with Taxol. Flow cytometry dot plot showing the gating of JC-1-aggregates and JC-1-monomer populations. Numbers are damaged cell population (percentage/total). Data are evaluated through at least three independent experiments.

To examine MST4′s function in breast cancer cell survival and chemo-sensitivity, we treated cells with the chemotherapeutic agent, Taxol (paclitaxel), which induces apoptosis and is commonly utilized for the treatment of breast cancer [31]. After Taxol treatment, we analyzed the level of cleaved caspase 3, which is an indicator of apoptosis in these cells. While wild type MST4 overexpression inhibited the level of cleaved caspase 3, expression of a kinase-defective MST4 mutant MST4/T178A restored Taxol-induced expression of cleaved caspase 3 (Figure 3a). Moreover, we performed Western blot of PARP cleavage in MDA-MB-231 cells with different expressing levels of MST4 (Figure 3b), exogenous expression of MST4 inhibited Taxol-induced PARP cleavage. In contrast, knocking down MST4 increased the Taxol-induced PARP cleavage in MDA-MB-231 cells. To further confirm the result, we measured mitochondrial membrane potential, which is another indicator for apoptosis. Mitochondrial membrane potential was examined utilizing flow cytometry (JC-1 dye), showing an increase in depolarized mitochondria, indicating increased apoptosis for MST4 knockdown cells, compared to MDA-MB-231 control cells (Figure 3c). Collectively, these results strongly support that MST4 plays a crucial role in promoting cancer cell survival and drug resistance. 

### 3.4. MST4 Regulates Epithelial-Mesenchymal Transition in Breast Cancer

**Figure 4:**
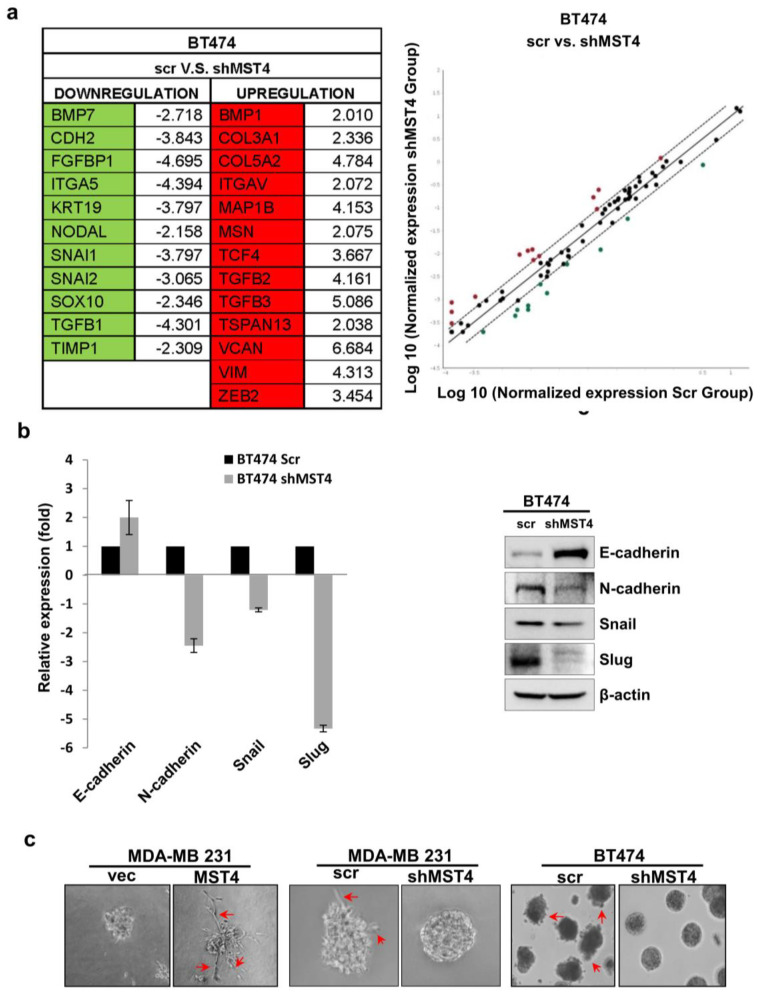
MST4 regulates epithelialmesenchymal transition (EMT) in cancer cells. Total RNA was isolated and used to synthesize cDNA for a real-time PCR-based EMT array. Data were analyzed through Qiagen software. (**a**) Using EMT qPCR array, BT-474 shMST4 cells were compared to their control for the expression of EMT associated genes. (**b**) EMT related genes such as E-cadherin, N-cadherin, snail, and slug were analyzed via qPCR and immunoblot in BT-474 MST4 knockdown cells. The bar diagram shows fold changes. β-actin was a loading control. Data are evaluated through at least three independent experiments. (**c**) Microscopic observation of an acinar-like structure with 3D culture in MST4 overexpression and knockdown MDA-MB-231 and BT-474 cells. The red arrows indicate acinar-like structures. Cells were observed and counted in ten random microscopic fields.

EMT is a biological conversion process in which epithelial cells lose cell polarity and are transformed to a mesenchymal phenotype. This phenomenon involves the loss of cell–cell adhesion, degradation of the underlying basement membrane, and the acquisition of migratory or invasive properties [32,33]. In previous results, knockdown of MST4 has shown inhibition of the migratory and invasive properties of cancer cells (Figure 2). Thus, we reasoned that MST4 may be involved in regulation of EMT. To test this hypothesis, we probed EMT-related genes that are regulated by MST4 using a qPCR array analysis for a panel of EMT markers in BT474 cells. Accordingly, the qPCR array showed that cells with MST4 knockdown resulted in an enrichment of epithelial markers but a decrease in mesenchymal markers, as compared to controls (Figure 4a). We further examined whether knockdown of MST4 could reverse EMT in BT-474 cells. To this end, qPCR and Western blotting were carried out for selected EMT regulating genes including E-cadherin, N-cadherin, snail, and slug. The qPCR (Figure 4b, left) and Western blot (Figure 4b, right) results clearly showed that MST4 knockdown can increase the expression of E-cadherin while reducing snail, slug, and N-cadherin expression as compared to control (Figure 4b). Moreover, we performed 3D culture assays to examine cellular invasiveness in MST4 overexpression and knockdown cells. As we anticipated, MST4 overexpressing MDA-MB-231 cells were observed to be more invasive as compared to control cells. In contrast, knockdown of MST4 in MDA-MB-231 and BT474 cells showed a less invasive phenotype compared to control cells in the 3D culture (Figure 4c). Morphologically, cells expressing a low level of MST4 formed typical breast acinar-like structures, whereas the cells overexpressing MST4 showed a more invasive phenotype with multiple invasive protrusions (red arrow) formed in the 3D structures (Figure 4c). Taken together, MST4 appears to be central as a key gene that is intimately involved in the EMT process.

### 3.5. MST4 Regulates AKT Pathway

**Figure 5:**
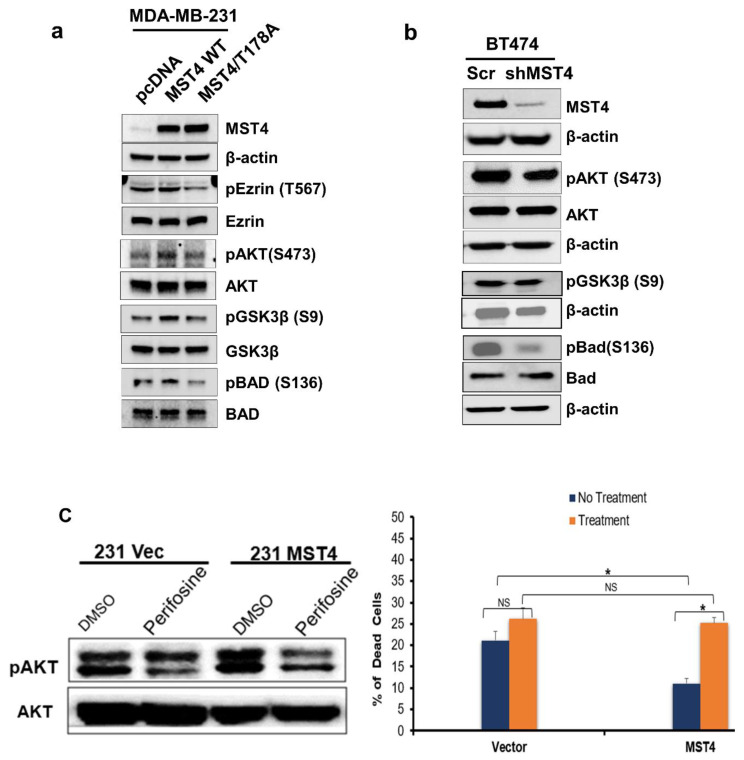
MST4 regulates AKT pathway. (**a**) MDA-MB-231 cells were transfected with pcDNA 3.1, MST4 wild type, and kinase defective mutant (T178A) MST4 plasmid vectors. After 48 h of transfection, the cell lysates were analyzed via immunoblot. Protein expression levels of MST4, pEzrin (Thr 567), Ezrin, pGSK3β (Ser 9), GSK3β, pAKT (Ser 473), AKT, pBAD (Ser 136), and BAD are shown; b-Actin served as the loading control. (**b**) BT474 cells were transfected with scramble sequence control and shMST4 to knockdown MST4 expression. After 48 h of transfection, the cell lysates were analyzed via immunoblot. Protein expression levels of MST4, pAKT (Ser 473), AKT, pGSK3β (Ser 9), pBAD (Ser 136), and BAD are shown; b-Actin levels served as the loading controls. (**c**) The control and MST4 high-expressing MDA-MB-231 cells were treated with Akt inhibitor perifosine; the cell death was detected by counting trypan blue stained and living cells. Cell lysates were analyzed via immunoblotting to measure the levels of pAKT (Ser 473) and total AKT. * indicates statistically significant (*p* < 0.05). N.S. indicates no statistical significance (*p* > 0.05).

MST kinases activate multiple signaling pathways including p38MAPK, c-Jun N-terminal kinase, ERK/MAPKs, and AKT in a cell-specific manner [24]. For example, in pituitary cells, MST4 activates p38MAPK and AKT pathways [34]. To investigate the cellular signaling pathways regulated by MST4 in breast cancer cells, we examined the levels of AKT in wild-type, and MST4 overexpressing MDA-MB-231 cells. Since MST4 is a protein kinase, we also investigated whether its activity is required for MST4 signaling during EMT and apoptosis by transfecting a kinase-defective (T178A) mutant in MDA-MB-231 cells (Figure 5a). Interestingly, cells with MST4 overexpression showed an increase in phosphorylation of MST4 substrate protein Ezrin, a protein activating cancer cell invasion, and GSK3β, a protein regulating EMT progression. In contrast, the phosphorylation of Ezrin and GSK3β was reduced in kinase-defective mutant MST4/T178A expressing cells (Figure 5a). Additionally, overexpressing MST4 increases phosphorylation of AKT and BAD, but the kinase-defective mutation of MST4 reduced AKT and BAD activation (Figure 5a), suggesting MST4 regulates cell survival though Akt/BAD signaling axis. Moreover, the activation of AKT and its downstream GSK3β and BAD was inhibited in MST4 knockdown BT474 cells (Figure 5b), suggesting MST4 is required for AKT and its downstream signaling pathway activation in breast cancer cells and is crucial for cancer cell survival and EMT via the MST4-AKT-BAD or MST-AKT- GSK3β signaling cascade. To further confirm this, we treated the control and MST4 high-expressing MDA-MB-231 cells with an Akt inhibitor perifosine (KRX-0401) [35]. The results indicate that perifosine effectively inhibited Akt activation, and blocked the MST4 overexpression mediated anti-apoptosis effect (Figure 5c), confirming that MST4 activated Akt is essential for cell survival.

### 3.6. TCGA and Patient Tissue Microarray Analyses of MST4 Expression

**Figure 6:**
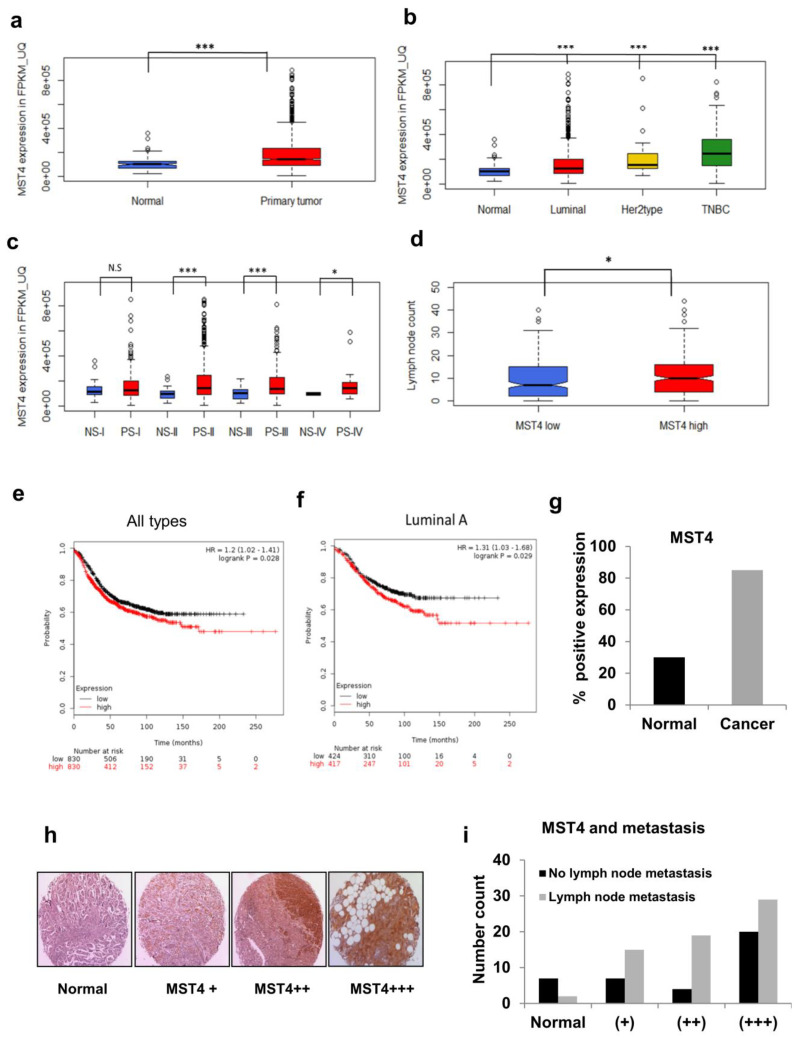
TCGA and Tissue Microarray analyses of MST4 expression. (**a**) TCGA BRCA Illumina high sequencing RNA seq data (level 3 gene expression data) for BRCA solid tumor and normal tissue samples. Gene expression data (in FPKM_UQ) were obtained for MST4 (NCBI Gene ID# 51765, Ensemble ID ENSG00000134602) for 1102 tumors and 113 normal solid tissue samples. Data were analyzed using the R program. (**b**) Level of MST4 expression was analyzed in different breast cancer types (luminal, HER2, TNBC) compared to normal. (**c**) MST4 expression was analyzed in different breast cancer stages I through IV, compared to non-tumor patient samples (NS). (**d**) MST4 levels associated with lymph node metastasis were evaluated in MST4 low and high breast cancer patient samples. (**e**) Survival plot was analyzed by the level of MST4 expression (low and high) in all types of breast cancer patient data. (**f**) Survival plot was analyzed by the level of MST4 expression (low and high) in luminal A type breast cancer patients. (**g**) Graph shows the % positive expression of MST4 in non-cancerous and cancer cases. (**h**) Images show different expression levels of MST4 in TMA breast cancer core samples. According to the staining scores, all the samples were stratified into four grades: no MST4 staining (Normal), weak MST4-positive staining (+), moderate MST4-positive staining (++), and strong MST4-positive staining (+++). (**i**) Graph shows lymph node metastasis associated with different MST4 expression levels. * indicates statistically significant difference in the expression of MST4 between normal and tumor tissues at *p* < 0.05 (*) and *p* < 0.01 (***). N.S. indicates no statistical significance (*p* > 0.05).

To study the clinical relevance of our in vitro findings, we analyzed the TCGA patient data sets. MST4 FPKM-UQ values were collected for solid normal tissues (n = 113), and primary tumors: luminal (n = 820), HER2 type (n = 37), and triple negative breast cancer (TNBC) (n = 115). After comparing median values, we found that MST4 gene expression is higher in primary tumors, compared to non-cancerous tissue (Figure 6a). Moreover, compared to the non-cancerous tissue, there is a 24% increase in MST4 gene expression for the luminal sub-type, a 52% increase in the HER2 type, and a 144% increase in triple negative breast cancer TNBC (Figure 6b). Furthermore, MST4 FPKM_UQ values were collected for Stage I-IV for solid normal tissue (NS-I (n = 20), NS-II (n = 65), NS-III (n = 25), NS-IV (n = 2)) and primary tumor tissue sample (PS-I (n = 182), PS-II (n = 627), PS-III (n = 249), PS-IV (n = 20)). MST4 gene expression was found to be higher in cancerous tissues, which was statistically significant for stage II, III, and IV breast cancer, but not for stage I (Figure 6c). By comparing median values for normal tissue with the stage of the tumor sample, we found that MST4 expression increased by 51% in stage II disease, by 32% in stage III, and by 46% for metastatic, stage IV breast cancer. In addition, we observed a 14% increase in MST4 expression for stage II cancer, compared to Stage I primary tumor samples (*p* = 0.001). Lymph node metastasis was also calculated for MST4 gene expression (low MST4 expression, n = 247/high MST4 expression, n = 246) and the high MST4 expressing group had a significantly higher number of lymph node metastases (*p* = 0.0135) as compared to the MST4 low expressing group (Figure 6d).

We then investigated whether there is a clinical correlation between levels of MST4 expression and patient outcome in breast cancer patients. Thus, we analyzed the global gene expression, relapse-free and overall survival data derived from GEO, TCGA, and EGA databases (the search was performed for STK26 (“224407_s_at”) on 12–20-2016 based on database release date (n = 4142)). As shown in Figure 6e, MST4 low (n = 830) and high (n = 830) patient groups were compared utilizing an online Kaplan–Meier plotter survival analysis. The result showed that patients with high MST4 expression had an overall poorer survival (hazard ratio = 1.20 with 95% confidence intervals, calculated log rank *p*-value = 0.028). Notably, the luminal sub-type patients, MST4 low (n = 424) and high (n = 417), showed the largest difference in overall survival among all patient groups (hazard ratio = 1.31 with 95% confidence intervals, calculated log rank *p*-value = 0.029) (Figure 6f). We also used a survival Kaplan–Meier plot for TNBC and HER2+ groups; however, the KM plot showed that these two groups had no statistical significance (*p* = 0.40 and 0.32 respectively). Of note, in this analysis MST4 expression was also higher in cancer cases compared to normal tissues (Figure 6g). These results strongly support that MST4 gene expression is associated with breast cancer progression, ultimately translating into a poorer overall survival for MST4 overexpressing patients. 

To validate the findings from TCGA dataset analysis, we performed a breast cancer patient sample tissue microarray (TMA) analysis for MST4 expression levels in non-cancerous and breast cancer tissues. Accordingly, MST4 staining was mainly found in the cytoplasm and around the nucleus of tumor cells. We observed immunohistochemical staining ranged from little to high levels of MST4 expression between different tumor tissues (Figure 6h). It also revealed that MST4 expression was significantly higher in cancer compared to non-cancerous tissue. Additionally, lymph node metastasis was also observed to be strongly correlated with MST4 positivity with 67% (63/93 cases) of cases positive for lymph node metastasis, with only 22% positive for MST4 negative cases (Figure 6i), suggesting a role of MST4 in breast cancer metastasis.

## 4. Discussion

MST4 kinase exhibits multiple roles in the regulation of signaling pathways that govern cell mitosis, homeostasis, polarity, migration, apoptosis, proliferation, and cellular differentiation [24,25,26,27,28,29]. MST4 has been reported to play a tumorigenic role in several cancer types, such as colorectal cancer [36], gastric cancer [37], glioblastoma [38], and hepatocellular carcinoma [26]. However, very little is known about the role and involvement of MST4 in breast cancer pathogenesis. In this study we provide, for the first time, evidence to show that MST4 promotes EMT via the MST4-AKT signaling pathway, together with its functions in cell proliferation, migration/invasion, cell survival, and chemoresistance, suggesting that MST4 may serve as a potential oncogene that can be exploited and targeted for breast cancer therapy.

A recent study of gastric cancer by Li et al. [37] reported that MST4 is correlated with a poor patient outcome, and promotes metastasis through activation of Ezrin mediated EMT regulating gene expression. Our findings are consistent with Li’s findings, and we extended Li’s finding to further explore the downstream signaling of Ezrin. We report here that MST4 activates Ezrin and, through Ezrin, activates Akt and its downstream molecules involved in cell survival and EMT regulation. These findings indicate that the MST4/Ezrin/Akt axis plays a crucial role in cancer cell EMT and metastasis.

In contrast to our findings, Dian et al. reported conflicting results in hepatocellular carcinoma cells. In their study, MST4 inhibits motility and invasive potential of hepatocellular carcinoma cells by inactivating the PI3K/AKT signaling pathway, which leads to the decrease of snail transcription factor, thus blocking EMT [39]. However, it is important to note that in most cancer types studied, the role of MST4 is in line with an oncogenic function, which is consistent with our report. Given the contrasting observations and the potential pro-tumor and antitumor effects of MST4, it is important to explore more the roles and its underlying mechanisms in different cancer types. Intriguingly, in the patient study we found that the highest MST4 expression appears in stage II cancer and a very high ratio of lymph node metastasis appears in MST4 (++) IHC expression. This leads to a hypothesis that MST4 is important in support of breast cancer cell survival for stage II tumors, and the moderate expression rather than overexpression of MST4 supports metastatic dissemination. This adds a layer of complexity to the role of MST4 in cancer progression, suggesting that different experimental models may have different impacts on the results obtained.

We used the online bioinformatic tool, “cBioPortal” (https://www.cbioportal.org/) (accessed on 10 December 2022), to analyze the correlation between MST4 and EMT related gene expression in breast cancer patient samples. We found that MST4 expression had weak correlation with several EMT associated genes such as N-Cadherin (*CDH2*), Sox10, FGFBP1, etc. (*p* < 0.05), while its expression also showed a weak reverse correlation with EMT associated gene such as Slug (SNAI2) (*p* < 0.05) (Supplementary Information). Thus, more in-depth bioinformatic studies are needed to clarify the correlation of MST4 and EMT related genes in large patient sample cohorts.

**Figure 7:**
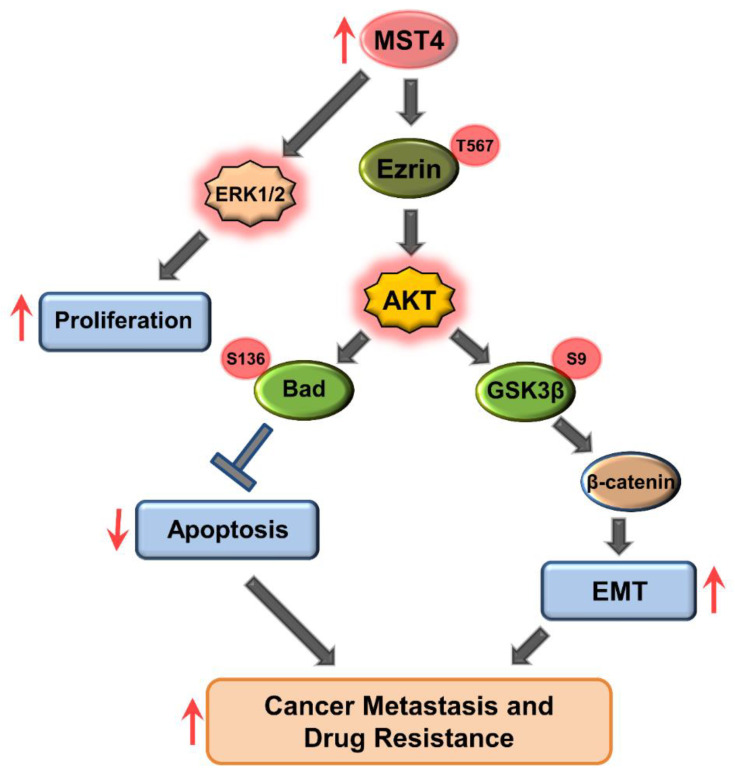
MST4 regulates EMT signaling via AKT and its downstream signaling pathway in breast cancer. Proposed model for MST4-mediated EMT signaling in breast cancer. Our study demonstrates that up-regulated MST4 leads to AKT signaling activation. This, in turn, promotes cell proliferation, migration, invasion, and EMT, and inhibits apoptosis pathways. ↑ indicates increased expression of MST4 enhances tumor cell proliferation, EMT and increased metastasis and drug resistance. ↓ indicates decreased tendency of tumor apoptosis. → indicates stimulation of downstream cascades and phenotypes. Ʇ indicates inhibition.

In summary, our study demonstrates that up-regulated MST4 promotes cell proliferation, migration, and invasion via the activation of Ezrin-AKT signaling pathways which in turn induces EMT transcription factors, such as snail, slug and N-cadherin, highlighting the central role of MST4 in regulation of EMT. On the other hand, MST4 activated Ezrin-AKT signaling leads to the phosphorylation of BAD which in turn increases the survival and chemoresistance of breast cancer cells (Figure 7). Collectively, our results suggest that MST4 may serve as a novel tumor promoting gene in breast cancer. 

## Data Availability

Data are contained within the article or Supplementary Material.

## Supplementary Materials

The following supporting information can be downloaded at: https://www.mdpi.com/article/10.3390/cells11244057/s1, Figure File S1.
