# Supplementary material for: MST4: A Potential Oncogene and Therapeutic Target in Breast Cancer
Source: Cells. 2022 Dec 15;11(24):4057. doi: 10.3390/cells11244057 (PMC9777386; doi:10.3390/cells11244057)
Supplement: Supplementary file 1 [file cells-11-04057-s001.zip › cells-2072422-supplementary.pdf]

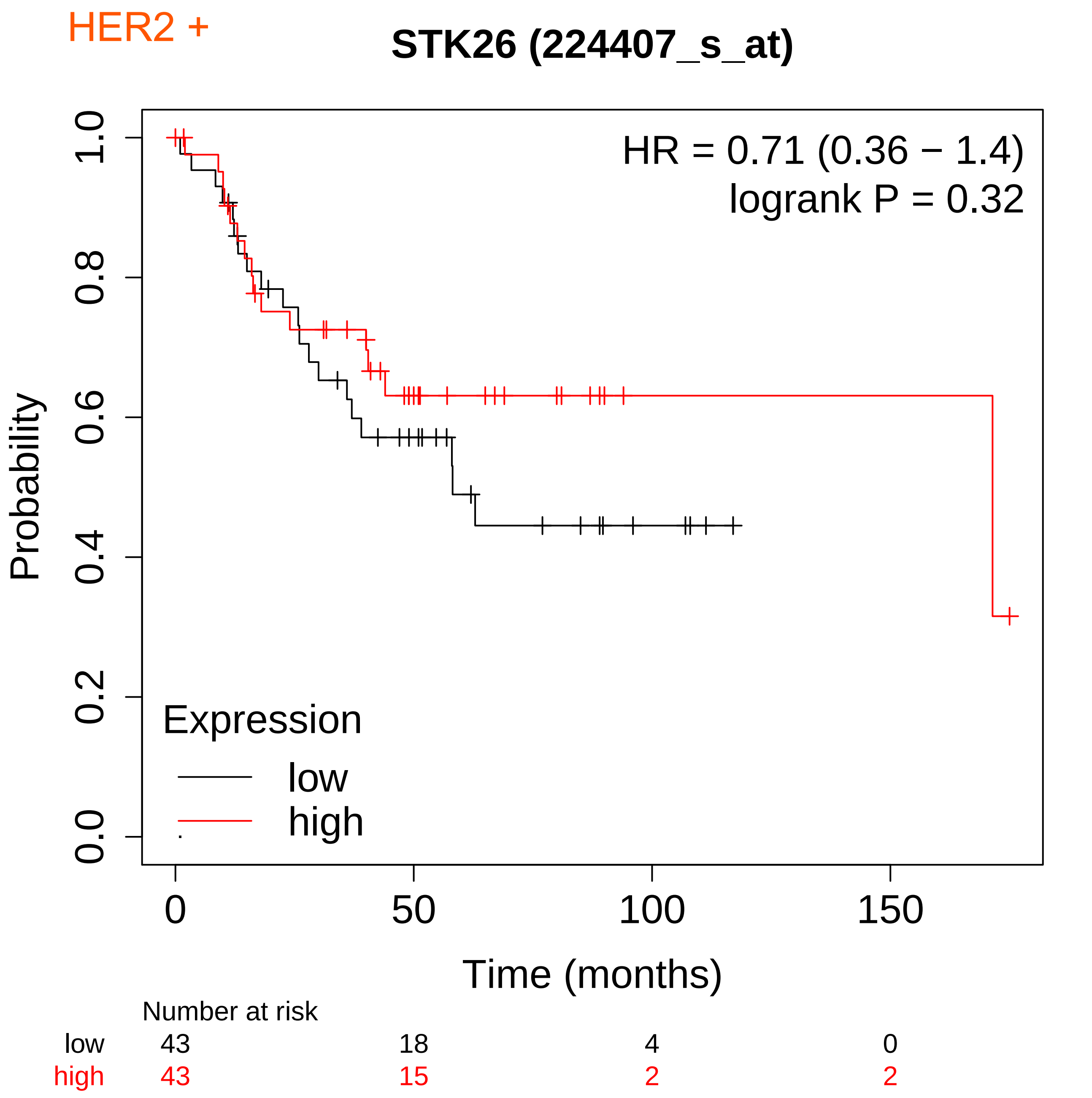

HER2 +
STK26 (224407_s_at)
HR = 0.71 (0.36 − 1.4)
logrank P = 0.32
Probability
0.0
0.2
0.4
0.6
0.8
1.0
Expression
low
high
0
50
100
150
Time (months)
Number at risk
low 43 18 4 0
high 43 15 2 2

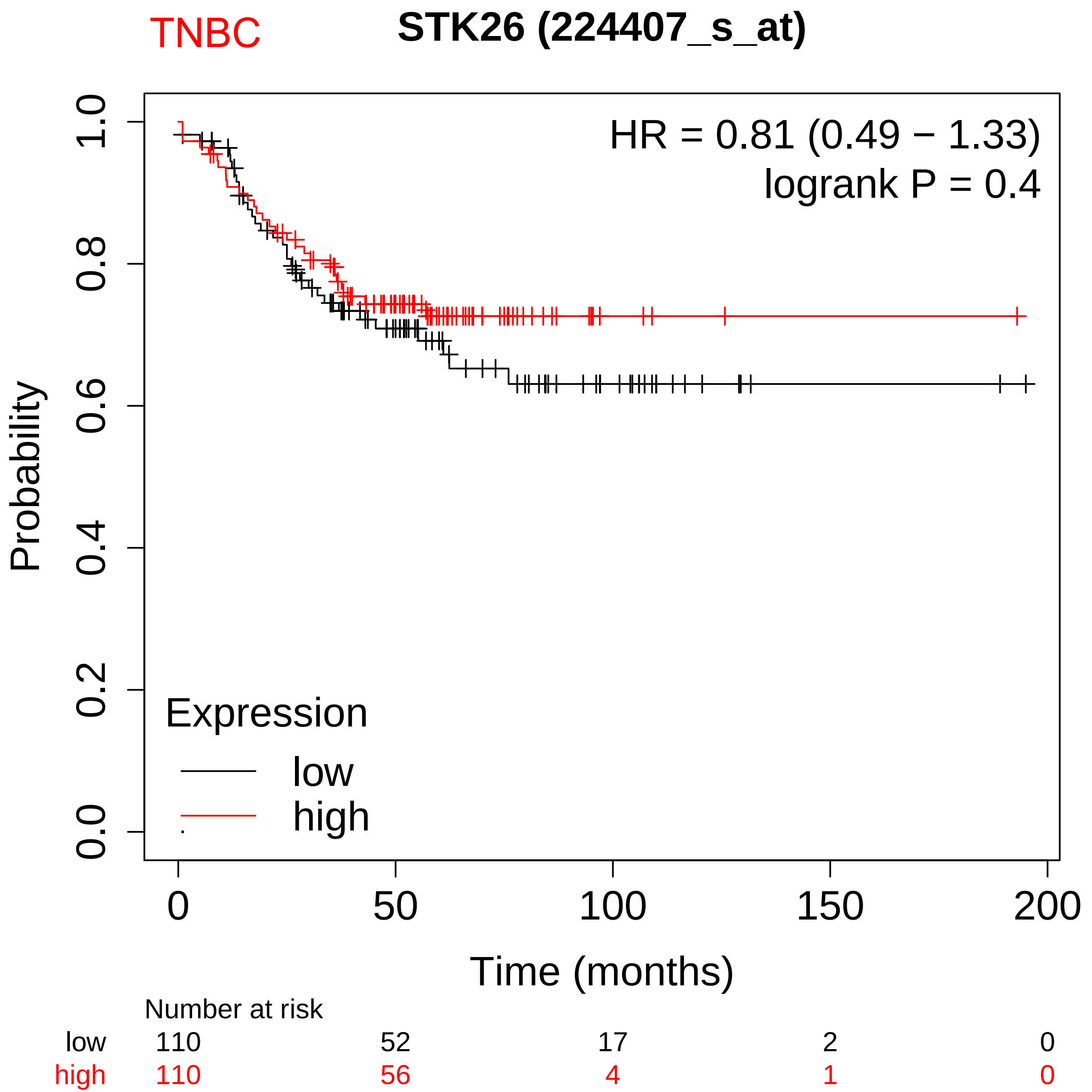

TNBC
STK26 (224407_s_at)
HR = 0.81 (0.49 − 1.33)
logrank P = 0.4
Probability
0.0
0.2
0.4
0.6
0.8
1.0
Expression
low
high
0
50
100
150
200
Time (months)
Number at risk
low 110 52 17 2 0
high 110 56 4 1 0

mRNA expression level of MST4 (aka STK26) and EMT related genes by microarray-Page 1

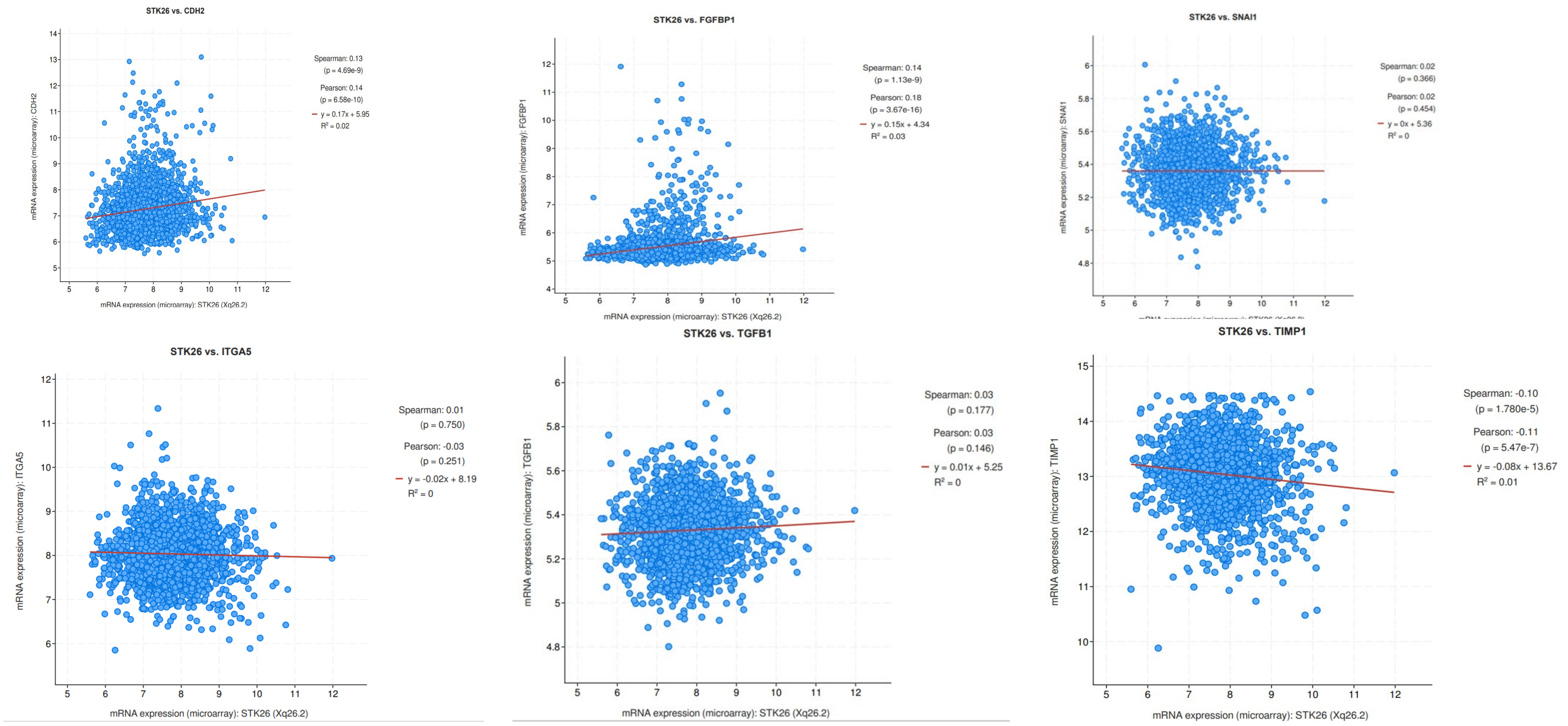

"cBioPortal" (https://www.cbioportal.org/), and Breast Cancer dataset (METABRIC, Nature 2012 & Nat Commun 2016)

mRNA expression level of MST4 (aka STK26) and EMT related genes by microarray-Page 2

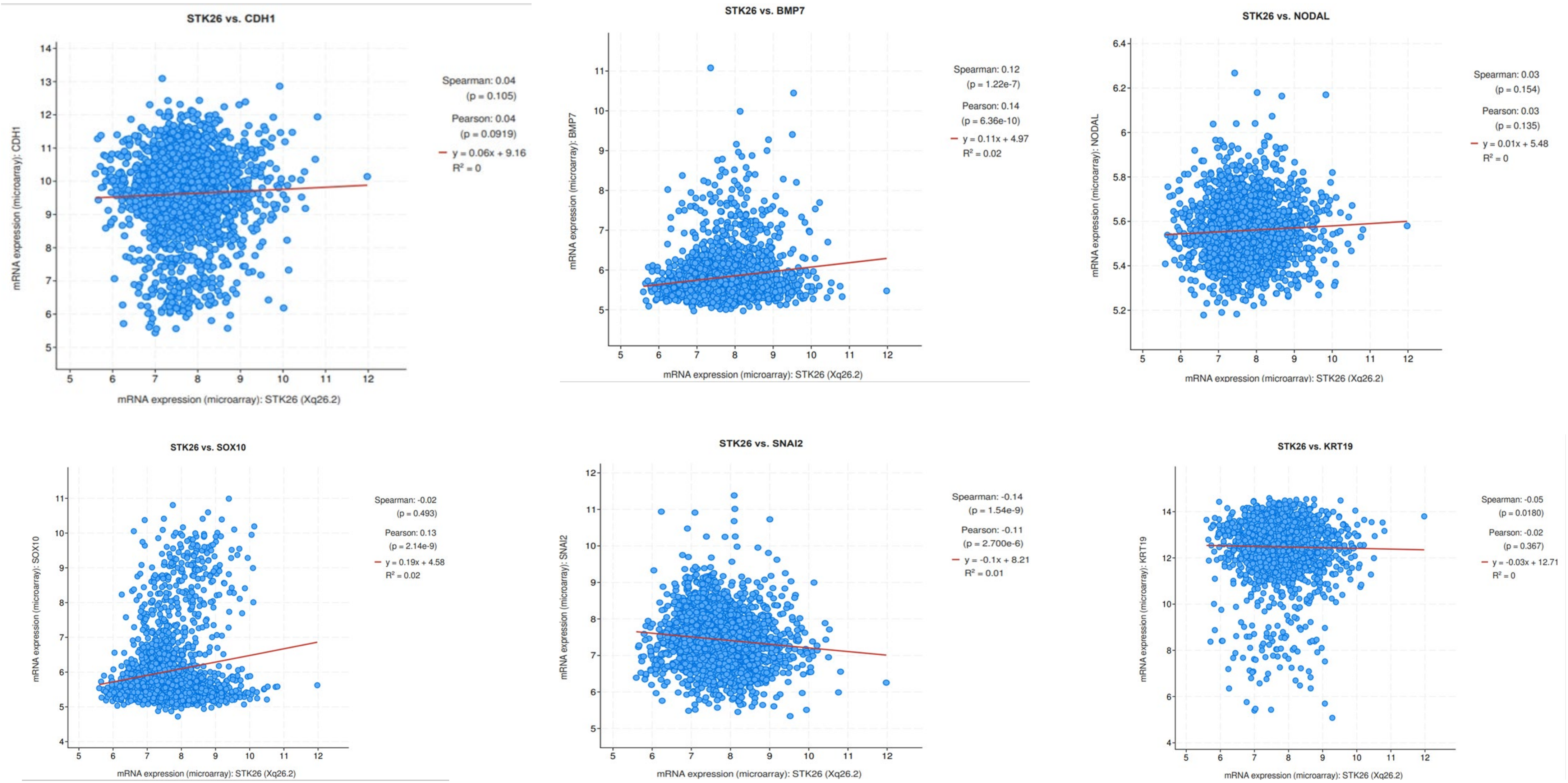

“cBioPortal” (https://www.cbioportal.org/), and Breast Cancer dataset (METABRIC, Nature 2012 & Nat Commun 2016)
